# A novel lesion severity index to predict 90-day postoperative survival in brain metastasis patients

**DOI:** 10.1007/s11060-025-05109-7

**Published:** 2025-06-11

**Authors:** Daniel C. Kreatsoulas, Joanne Kim, Mark Damante, Anna Orr, Joshua Wang, Joshua Vignolles-Jeong, Maxwell Gruber, Varun Shah, Nicholas Musgrave, Russell Lonser, Daniel Prevedello, J. Bradley Elder, Douglas A. Hardesty

**Affiliations:** 1 Department of Neurological Surgery, The Ohio State University Wexner Medical Center, N1019 Doan Hall, 410 West 10th Avenue, Columbus, OH 43210 USA; 2 Department of Biomedical Informatics, The Ohio State University, Columbus, OH USA; 3 The Ohio State University College of Medicine, Columbus, OH USA; 4 Department of Neurological Surgery, Case Western Reserve University Hospitals, Cleveland, OH USA; 5 Department of Neurological Surgery, St. Louis University Hospitals, St. Louis, MO USA; 6 Department of Neurological Surgery, Creighton University Hosiptals, Omaha, NE USA

**Keywords:** Brain metastasis, Prognostication, Risk assessment, Survival

## Abstract

**Introduction:**

Brain metastasis patients require granular surgical risk evaluation. The authors aimed to improve prognostication for brain metastasis patients by creating the Metastatic Brain Lesion Score (MBLS).

**Methods:**

This is a retrospective cohort study of patients undergoing craniotomy for brain metastasis between Jan 1, 2014 and Sept 30, 2021 at a large tertiary referral center. Patients were excluded if they had craniotomy for non-metastatic lesions, radiation necrosis, or skull metastases only. The primary outcome was to define factors that predicted mortality at 90 days. Secondary measures were mFI-11 and RPA classifications. Multivariable logistic regression analyses were conducted to identify the clinically and statistically relevant predictors for the predicted mortality at 90 days with the creation cohort (*n* = 548). The metric developed was validated with the new data (validation cohort, *n* = 318), and was compared its prediction performance with secondary measures.

**Results:**

866 consecutive patients (*n* = 548 creation cohort, *n* = 318 validation cohort) were reviewed and analyzed. Score factors included in the MBLS were: age > 65 (OR 1.97 (95%CI 1.19–3.02)), presence of supra- and infratentorial metastases (OR 1.696 (1.04–2.78)); hemorrhagic metastasis (OR 1.699 (1.09–2.64)); chronic opiate use (OR 2.34 (1.43–3.84)); poor functional status (OR 1.90 (1.22–2.97)); and presence of deep brain/brainstem lesions (OR 2.01 (1.07–3.78)). 61/164 (37.2%) creation cohort patients and 51/116 (43.9%) validation cohort patients with MBLS score ≥ 3 were deceased at 3 months. High-risk patients in the creation cohort were significantly more likely to be deceased 3 months postoperatively (OR 3.699 (95%CI 2.41–5.682), *P* < 0.001). In the validation cohort, MBLS was highly predictive (OR 5.311 (95%CI 3.06–9.22), *P* < 0.001) with a c-statistic of 0.696.

**Conclusions:**

The MBLS provides “high-risk” surgical categorization for brain metastasis patients. By using preoperative characteristics obtainable from imaging and chart review, it can be utilized in preoperative discussions, giving a clearer view of potential postoperative course and outcome.

**Supplementary Information:**

The online version contains supplementary material available at 10.1007/s11060-025-05109-7.

## Highlights

Brain metastasis patients require unique surgical risk stratification because they have different risks than glioma or benign craniotomy patients.

The Metastatic Brain Lesion Score takes into account both clinical and imaging factors to help surgeons and oncologists predict death 90 days after craniotomy.

This quickly calculable score will assist with preoperative risk stratification and discussion with patients to improve patient care and team communication.

**Supplementary Information:**

The online version contains supplementary material available at 10.1007/s11060-025-05109-7.

## Introduction

Risk reduction is an important area of healthcare progress; major nationwide programs have addressed catheter-related urinary tract infections, central line infections, and prevention of deep venous thrombosis, amongst others. Surgical risk scores aid in risk reduction via adequate patient selection, preoperative medical optimization, and general information for families and patients to know before consenting for a procedure. Several surgical risk scores already exist, such as the modified frailty index-11 (mFI-11) [1–5], American Society of Anesthesiology (ASA) score [6, 7] and the National Surgical Quality Improvement Program-21 (NSQIP) calculator [8]. These are based on general health but not necessarily surgical disease-specific characteristics. Sometimes when using these scores, even “high risk” patients undergo surgery, and “low risk” patients may do poorly after surgery.

Although existing scores have been applied to, and published regarding, perioperative risk after craniotomy [1, 9] determining individual risk for neurosurgical procedures remains difficult. Brain metastases are common, with an estimated 20% or more of all patients with cancer being diagnosed with a brain metastasis during their treatment course and 3–5% of these patients requiring craniotomy for tumor resection [10–12]. Risk estimation in brain metastasis is particularly difficult because while a cancer patient may have few traditional medical comorbidities, they may be high risk based on their primary cancer or brain metastases characteristics. As such, we conducted a large single-institution retrospective cohort analysis of surgical brain metastasis patients to create a novel brain metastasis severity score (the Metastatic Brain Lesion Score [MBLS]) for prediction of postoperative mortality at 90 days.

## Methods

This retrospective cohort study was approved by local institutional review board [IRB# 2019H0446]. All patients who underwent craniotomy at a single tertiary referral center from 1/1/2014 to 10/31/2019 were identified via operating room case logs (utilizing CPT codes 61510 and 61519), then all non-metastatic tumors were excluded manually. This initial “creation” cohort consisted of 548 patients. Chart review was performed, obtaining demographics, past medical and surgical history, cancer diagnosis and treatment history (primary histology, timeline of therapy, types and use of therapies, prior lines of treatment if any, time of diagnosis and discovery of brain metastasis), brain tumor characteristics (size, number, volume, locations, presence of hemorrhage, proximity to critical brain structures), clinical presentation prior to craniotomy (neurological exam, Karnofsky performance status based on expert evaluation, non-surgical problems during preoperative course), and postoperative course. The primary outcome was death within 90 days of initial surgery. Patients that had multiple craniotomies were considered as separate if the second craniotomy was on a separate metastasis or if the repeat happened > 90 days after the first. If patients had multiple craniotomies under the same anesthesia, they were considered as a single case identifier.

Statistics were performed via SPSS version 28 (IBM, Armonk, NY, USA) and R software (R 4.4.1). Continuous variables were analyzed via t-test and linear regression analysis, and categorical variables were analyzed via the chi-square test, the Fisher’s exact test, and single and multivariable logistic regression analysis. Significance was determined as *p*-value < 0.05. After all potential score factors were analyzed, those that were significant entered a multivariable model to evaluate the association with death at 90 days. This was performed in a stepwise fashion to determine score variables. Point values were assigned to the score based on estimated parameter beta coefficients as described in other scoring systems [13].

The created score was then evaluated via regression analysis, Kaplan-Meier curve creation, and receiver operating characteristic (ROC) c-statistic evaluation [13] to determine its association with death at 90 days. The strength of this score was also compared to the mFI-11 and the recursive partitioning analysis (RPA) [14] scores in the creation cohort. Finally, a second unique group of patients was obtained who had surgery at our institution for brain metastases from 11/1/2019 to 11/1/2021 (the “validation cohort”). This group of 318 patients was analyzed in the same manner as the creation cohort.

## Results

Demographics for both cohorts appear in Table 1. In the creation cohort, average age at surgery was 59.3 years (± 11.8 SD), 171 patients were older than 65 (31.2%), patients were 51.8% male, and 90.3% Caucasian. When comparing patients alive vs. deceased at 90 days, there was no significant difference in sex or race, but there were more patients older than 65 who were deceased at 90 days (*p* = 0.03). In the validation cohort, average age was 60.8 years (± 11.8 SD), 50% of patients were male, and 92.1% Caucasian. Compared to the creation cohort, the validation cohort contains larger proportion of African American (AA) population in deceased group. There was no significant difference in the percentage of patients deceased at 90 days in the creation cohort vs. the validation cohort (20.8% vs. 24.2%, *p* = 0.243). A full breakdown of malignancy characteristics appears in Supplementary Table 1. The most common primary malignancy in both cohorts was non-small cell lung cancer, followed by melanoma, renal cell carcinoma, and breast cancer. Neither cohort showed significant difference in tumor volume between groups, but significant differences appeared in other tumor characteristics later analyzed for the MBLS (Table 2).

**Table 1:** Demographics of the creation and validation cohorts. SD = standard deviation

**Creation Cohort**			
**Characteristic**	**No. (%, range)**		***p*** **-value**
	**Alive > 90d**	**Deceased at 90d**	
**Age at Surgery (mean)**	58.8 (11.8 SD)	61.3 (11.9 SD)	0.053
Less than or equal to 65	308/434 (71.0%)	69/114 (60.5%)	**0.03**
Older than 65	126/434 (29.0%)	45/114 (39.5%)	
**Sex**			
**Male**	221/434 (51%)	63/114 (56%)	0.461
**Female**	213/434 (49%)	51/114 (44%)	
**Race**			
**White**	392/434 (90%)	103/114 (91%)	0.719
**African American**	36/434 (8%)	8/114 (7%)	
**Other**	8/434 (2%)	3/114 (2%)	
**Validation Cohort**			
**Characteristic**	**No. (%**, **range)**		***p*** **-value**
	**Alive > 90d**	**Deceased at 90d**	
**Age at Surgery (Mean)**	60.6 (11.7 SD)	61.4 (11.4 SD)	0.602
Less than or equal to 65	161/241 (66.8%)	46/77 (59.7%)	0.258
Older than 65	80/241 (33.2%)	31/77 (40.3%)	
**Sex**			
**Male**	117/241 (48.5%)	42/77 (54.5%)	0.359
**Female**	124/241 (51.5%)	35/77 (45.5%)	
**Race**			
**White**	227/241 (94.2%)	66/77 (85.7%)	**0.021**
**African American**	10/241 (4.1%)	10/77 (13.0%)	
**Hispanic**	0	0	
**Asian**	4/241 (1.7%)	1/77 (1.3%)	

**Table 2:** Frequencies of chosen tumor characteristics among patients included in the creation and validation cohorts

Tumor Characteristics		Creation Cohort		Validation Cohort	
		Alive > 90d	Deceased at 90d	Alive > 90d	Deceased at 90d
Intracranial Tumor Burden (mL) (mean, SD)		18.8 (± 27.5)	16.5 (± 19.6)	20.4 (± 20.2)	20.3 (± 15.7)
		*P* = 0.552		*P* = 0.122	
Presence of deep brain nucleus/brainstem lesions (No., %)		36/434 (8.3%)	21/114 (18.4%)	19/241 (7.9%)	16/77 (20.8%)
		*P* = 0.002		*P* = 0.002	
Presence of hydrocephalus or ventricular involvement (No., %)		68/434 (15.7%)	29/114 (25.4%)	41/241 (17.0%)	27/77 (35.1%)
		*P* = 0.015		*P* = < 0.001	
Presence of hemorrhage in metastasis (No., %)		198/434 (45.6%)	69/114 (60.5%)	98/241 (41.1%)	55/77 (71.4%)
		*P* = 0.005		*P* = < 0.001	
Location of metastases (No., %)	Supratentorial	303 (69.8%)	66 (57.9%)	158 (65.6%)	38 (49.4%)
	Infratentorial	42 (12.2%)	10 (8.8%)	33 (13.7%)	2 (2.6%)
	Both	69 (15.9%)	38 (33.3%)	49 (20.3)	37 (48.0%)
	*P* value	*P* = 0.015	*P* = < 0.001		

The association between different patient characteristics and 90-day mortality after brain metastasis surgery is shown in Table 3. First, we performed a simple logistic regression with each predictor and 90-day postoperative mortality. The result is shown in the ‘Univariable Analysis’ column in Table 3. Statistically significant association with 90-day mortality was found with age at surgery (*p* = 0.033), reduced functional status (*p* < 0.001), use of chronic pain medicine (*p* < 0.001), presence of deep brain nucleus/brainstem lesions (*p* = 0.002), presence of hydrocephalus or ventricular involvement (*p* = 0.016), presence of hemorrhage in metastases (*p* = 0.005) and the location of metastases (*p* = 0.004). We performed a stepwise multivariable logistic regression to identify the final predictor set of the risk score. The result is summarized in the ‘Multivariable Analysis’ column in Table 3. When these variables were analyzed together, the presence of deep brain nucleus/brainstem lesions and the presence of hydrocephalus or ventricular involvement (*p* = 0.059 and *p* = 0.094, respectively) were not statistically significant, but only the presence of hydrocephalus or ventricular involvement was dropped from the final model based on the evaluation of beta coefficients, *p*-values and clinical importance. Final multivariable model and the assigned score are shown in Table 4. Of note, based on beta coefficients and strength of correlation, similar to other reports of scoring system creation [13] chronic opioid use was given two points rather than one like the other factors.

**Table 3:** Univariable and multivariable logistic regression analysis results

Variables	Univariable Analysis		Multivariable Analysis	
	OR (95% CI)	*p*-value	OR (95% CI)	*p*-value
**Age at Surgery**				
Less than or equal to 65	Reference	0.033	Reference	0.007
Older than 65	1.594(1.038,2.448)		1.897(1.192,3.020)	
**Use of chronic pain medicine**				< 0.001
No	Reference	< 0.001	Reference	
Yes	2.193(1.381,3.482)		2.380(1.445,3.922)	
**Reduced functional status**				0.006
No	Reference	< 0.001	Reference	
Yes	2.354(1.539,3.600)		1.880(1.202,2.942)	
**Tobacco use**				
No	Reference	0.299		
Yes	0.800(0.524,1.220)			
**Presence of deep brain nucleus/brainstem lesions**				
No	Reference	0.002	Reference	0.059
Yes	2.496(1.393,4.475)		1.854(0.976,3.522)	
**Presence of hydrocephalus or ventricular involvement**				
No	Reference	0.016	Reference	0.094
Yes	1.836(1.120,3.011)		1.581(0.925,2.701)	
**Presence of hemorrhage in metastasis**				
No	Reference	0.005	Reference	0.016
Yes	1.828(1.200,2.783)		1.726(1.108,2.686)	
**Location of metastases**				
Supratentorial or Infratentorial	Reference	0.004	Reference	0.046
Both	1.938(1.231,3.051)		1.656(1.008,2.719)	
**Brain metastasis as an initial cancer diagnosis**				
No	Reference	0.606		
Yes	0.882(0.548,1.421)			

CI = confidence interval

**Table 4:** Final scoring justification for MBLS based on beta coefficients

Variables	Adjusted OR (95% CI)	*p*-value	Beta coefficient	Risk score assigned
**Age at Surgery**				
Less than or equal to 65	Reference	**0.004**	Reference	0
Older than 65	1.969(1.242,3.121)		0.677	1
**Use of chronic pain medicine**				
No	Reference	**< 0.001**	Reference	0
Yes	2.338(1.423,3.841)		0.849	2
**Poor functional status**				
No	Reference	**0.005**	Reference	0
Yes	1.900(1.216,2.969)		0.642	1
**Presence of deep brain nucleus/brainstem lesions**				
No	Reference	**0.031**	Reference	0
Yes	2.009 (1.068,3.779)		0.697	1
**Presence of hemorrhage in metastasis**				
No	Reference	**0.018**	Reference	0
Yes	1.699(1.093,2.640)		0.530	1
**Location of metastases**				
Supratentorial or Infratentorial	Reference	**0.036**	Reference	0
Both	1.696(1.035,2.779)		0.528	1

CI = confidence interval; OR = odds ratio

**Fig. 1:**
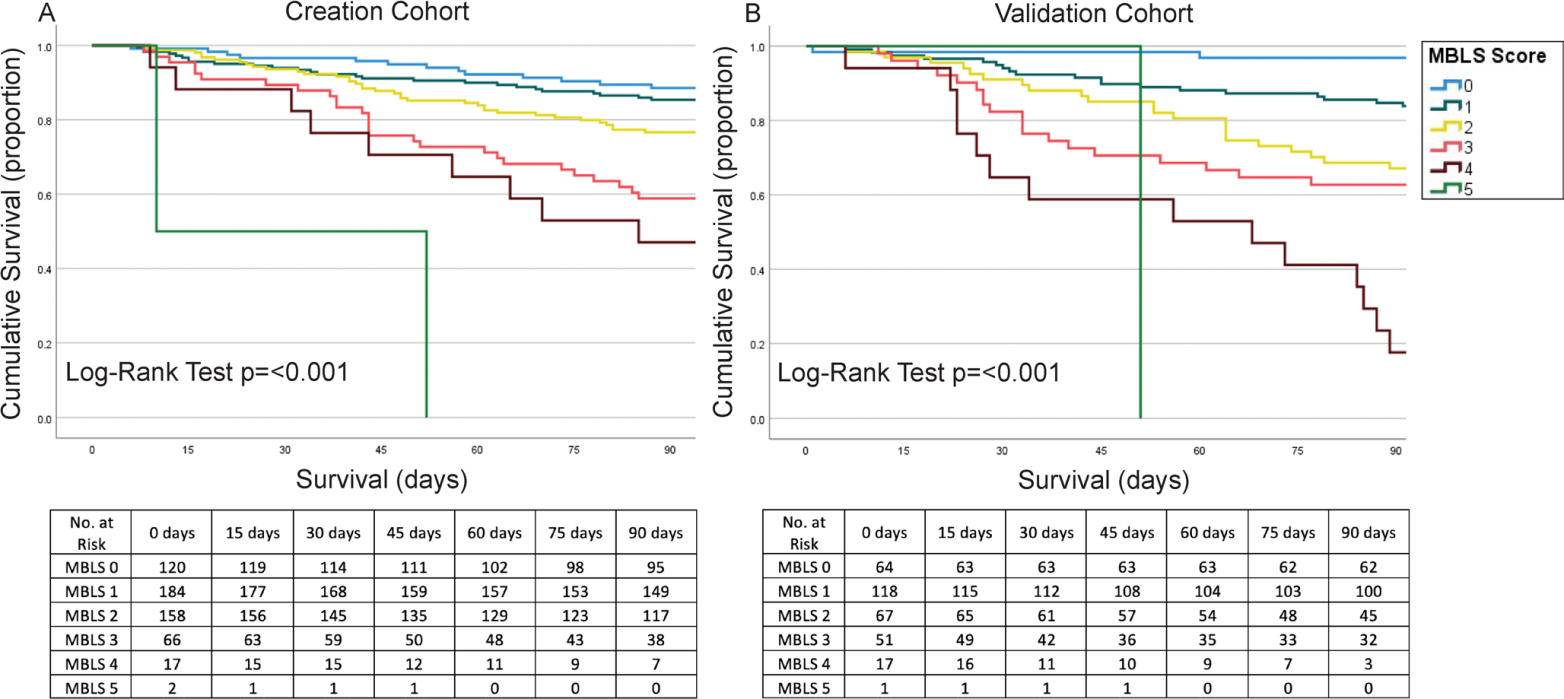
Kaplan-Meier curves depicting the stepwise increase in mortality with each additional point on the MBLS score. Listed below are life tables denoting the number of patients at risk at each time point. Legend for both listed to the right. 1 A = creation cohort; 1B = validation cohort

Scoring and analysis for the creation cohort appears in Supplementary Table 2. Most patients scored 0 (*n* = 81/548, 14.8%), 1 (*n* = 170/548, 31.0%), 2 (*n* = 133/548, 24.3%), or 3 (*n* = 93/548, 17.0%) on the MBLS. Scores of 4 (*n* = 52/548, 9.5%), 5 (*n* = 16/548, 2.9%), 6 (*n* = 2/548, 0.4%), and 7 (*n* = 1/548, 0.2%) were rarer. A significant increase in mortality at 90 days was noted between a score of 2 and 3 (15.8% vs. 30.1%) and a score of 3 and 4 (30.1% vs. 42.3%). To evaluate different scoring cutoffs, two analyses were performed, the first with a score of ≥ 3 being “high risk”, then with a score of ≥ 4 being “high risk”. The first analysis resulted in an OR of 3.70 (95% CI 2.41–5.68, *p* = < 0.001) for death at 90 days in the high-risk (score ≥ 3) group. The second analysis, scoring “high-risk” at a cutoff of ≥ 4, resulted in an OR of 4.25 (95% CI 2.51–7.17, *p* = < 0.001) for death at 90 days. A c-statistic analysis resulted as 0.649 for ≥ 3 and 0.601 for ≥ 4, suggesting improved performance of the ≥ 3 cutoff which was chosen as the final score. After creating this score, the validation cohort was collected, scored, and analyzed identically. Final evaluation of the model via Kaplan-Meier analysis revealed a significant difference in survival curves in the creation cohort for patients with a ≥ 3 MBLS (“high risk”) and a 0–2 MBLS, with high-risk patients having significantly worse overall survival at 90 days (Fig. 1). For the validation cohort, scoring results also appear in Table 5. When stratifying into score ≥ 3 as “high-risk”, regression analysis showed an OR of 5.31 (95% CI 3.06–9.17, *p* = < 0.001) for death at 90 days. Cohorts were similar in the percentage of patients deemed “high risk” for a ≥ 3 cutoff (30.0% vs. 36.5%). Survival analysis again revealed high-risk patients have a decrease in survival at 90 days (Fig. 1). The c-statistic was 0.691, indicating good predictive performance.

For comparison purposes, the mFI-11 was calculated for each patient (Supplementary Tables 3 and 4) and analyzed with the primary outcome. Using the mFI characteristics, patients were determined to be low or high frailty (scores of 0–2 or 3 + on the mFI-11). The proportion of patients deceased at 90 days increased as frailty category increased. This was also performed for RPA classification [14] and a similar pattern appeared, with proportion deceased at 90d increasing with increasing RPA classification severity (Supplementary Table 2).

**Fig. 2:**
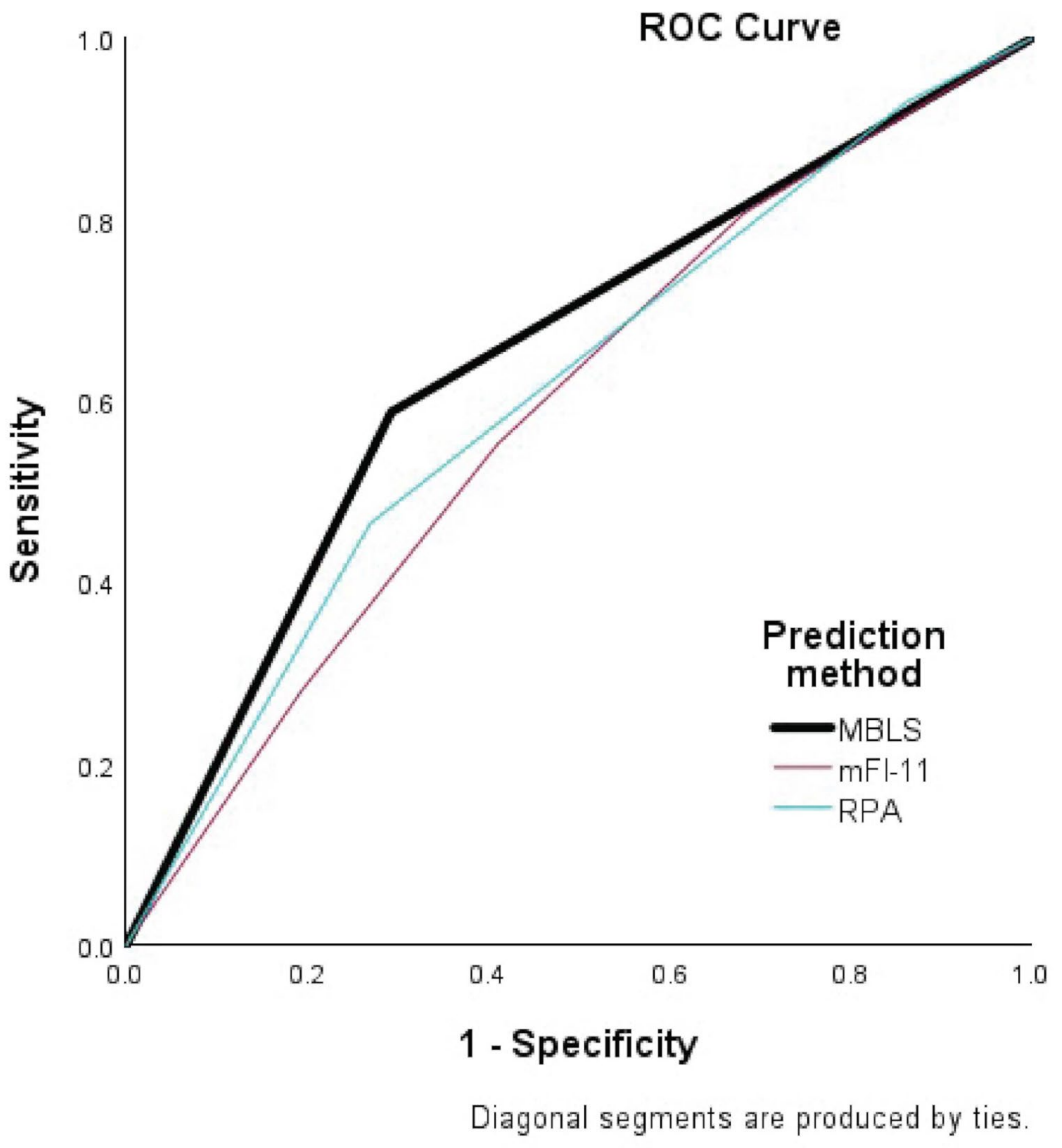
Graphical comparison of the area under the curve of the ROC statistics for MBLS Score 3 or more, high-frailty classification mFI-11, and RPA class 3 comparing their predictiveness for death at 90 days

To further illustrate differences in the scores, we directly compared the results of the MBLS in the creation cohort to the mFI-11 and the RPA classification (Supplementary Table 4, Fig. 2). The ROC curves of the MBLS, mFI-11 and RPA classification are shown in Fig. 2. The MBLS shows higher c-statistics (0.648), compared to other two metrics ( RPA = 0.609,mFI-11 = 0.514). We also conducted the DeLong test to compare the ROC curve of MBLS with ROC curves of mFI-11 and RPA metrics. The R software (R 4.4.1) and the R package ‘pROC’ was used for the DeLong’s test. The test result comparing the MBLS with mFI-11 was statistically significant (*p* < 0.001), but the test between MBLS and RPA was not ( *p* = 0.17) (Fig. 2, Supplementary Table 4). Accordingly, the MBLS suggests a trend toward better predictive performance than RPA classification with a slightly higher c-statistics, and is statistically significantly better than the mFI-11. Also the MBLS showed improved negative predictive value and sensitivity with comparable specificity to the other two scores. It did not label more patients high risk than the RPA classification, and did not lead to more “false negatives”, with a smaller percentage of low-risk patients deceased at 90 days than the mFI-11 or RPA classification.

## Discussion

Surgical risk stratification is an important part of modern surgical practice. It allows for more intricate discussion of risks and benefits and is crucial to informed consent. The MBLS accomplishes this for brain metastasis resection in a cohort of heterogeneous, diverse patients already selected for surgery by senior neurosurgeons. By using all-comers in its creation, the MBLS score’s intended purpose is to provide a general discussion and evaluation tool for any brain metastasis considered for surgery. It offers patient risk assessment based on both medical and imaging-related characteristics to give a more comprehensive picture of a patient’s potential outcome after surgery. Additionally, it was created in only brain metastasis patients, improving the direct applicability. Our score was validated in two cohorts that were similar demographically, in baseline health status, and in mortality outcomes. Additionally, the histological breakdown was consistent with epidemiological estimates [10, 15], suggesting the study sample is generalizable to the greater brain metastasis population.

Most other available scores offer stratification based on purely medical factors and are not isolated to brain metastases. For example, Youngerman and colleagues published an analysis of primary and metastatic craniotomy patients from the NSQIP database and calculated mFI-11 and ASA scores. Their analysis showed mFI-11 was more predictive than the ASA score for mortality, complications, prolonged length of stay, and unfavorable discharge at 30 days [9]. These results improved when the models were combined. Their model, while informative, may be time-consuming to calculate and complicated to interpret. We sought to create the MBLS in order to improve interpretation of patient risk and easily describe this interpretation to patients or non-neurosurgeons during a plan of care or tumor board discussion. We demonstrated that the MBLS outperforms both the mFI-11 and the RPA classification in our cohort, and believe that it provides a more patient-specific risk analysis than other risk scales.

Presence of hemorrhagic metastases was an important finding in our analysis. A point was given if there was hemorrhage in any metastasis, not necessarily the operative one. Hemorrhage can be a potential indicator of poor postsurgical outcome, as in massive hemorrhagic metastases that progress to obtundation and require emergency surgery. However, the vast majority of the hemorrhages in our cohort were non-emergent in nature, most often described as microhemorrhage in radiology reports. Hemorrhage into a brain metastasis is indicative of higher risk of leptomeningeal disease (LMD) [16] as well as longer hospital stay and increased cost [17]. Moreover, intracranial hemorrhage of any kind in cancer patients is associated with poor outcomes [17, 18] indicating that the addition of this factor to our score is warranted.

Two other unique factors we identified as independently associated with patient death within 90 days of surgery were the presence of both supratentorial and infratentorial metastases, and presence of deep brain nucleus/brainstem lesions. The first refers to patients who have an operative metastasis in the supratentorial space but non-operative lesions in the infratentorial space, and vice versa. Deep brain/brainstem metastases included thalamic and basal ganglia metastases as well as any midbrain, pontine, or medullary lesion. The literature for tumor location effect on radiation therapy is robust [19–21] but there is little discussion of the effect of multiple tumor locations on surgical outcome. There is even contention over the surgical management of patients with multiple brain metastases. One series of 32 patients with multiple brain metastases determined that the best outcomes were present in patients with 1–3 total metastases and a good functional status, and argues that patients with ³4 metastases would not benefit from surgery [22]. One explanation for this is: with a greater number of metastases, there is a higher likelihood of one being located in high-risk or eloquent areas [23]. Moreover, a location in the posterior fossa suggests higher risk for increased intracranial pressure and development of hydrocephalus, both of which portend worsened prognosis in metastatic disease [24]. This justifies inclusion in our scoring system. However, patients can benefit from an aggressive surgical approach if they are amenable and have a strong oncologic support team. With an emphasis on multidisciplinary care and careful discussions at tumor boards, patients with multiple metastases can benefit from surgery if there is a dominant symptomatic metastasis, if the patient has reasonable systemic disease control, and there are therapeutic options after surgery.

Functional status (KPS < 70) and age (> 65 years) had a significant impact on the MBLS score, mirroring the literature for other scores, where they are important in predicting chemotherapy candidacy [25–27] as well as outcome after many different procedures [28–33]. KPS has been used as a prognostic factor for brain metastases from various cancer types [34–36] and is present in other scoring systems for predicting survival including the RPA classification [14]. KPS also has better inter-rater reliability than the Eastern Cooperative Oncology Group score and the Palliative Performance Scale [37]. Finally, chronic preoperative opiate use has been demonstrated as a negative predictor for postsurgical patients in terms of complications, revision rates, and overall morbidity and mortality [38–42]. Chronic opioid use had a major impact on our score, with patients receiving two points if they met criteria. These clinical factors were important for rounding out the MBLS score.

The MBLS score provides a rapidly calculable set of criteria that can serve as a prognosticator for a brain metastasis patient’s mortality risk within 90 days of a craniotomy. The score factors implicate metastasis characteristics and imaging findings that are already considerations for the neurosurgeon when planning a case, as well as two important clinical factors. Other risk scales heavily weigh underlying medical condition, and few take into consideration disease-specific factors. After evaluating the mFI-11 and RPA classification in this cohort, the most risk-contributory factor is poor functional status; one of the reasons for inclusion in the MBLS score. Interestingly, there is not a significantly increased weight for functional status compared to the other factors in the MBLS based on odds ratios. Chronic opioid use is the most highly weighted factor, likely related to the general health status that a cancer patient requiring chronic pain medication would possess. Many other comorbidities and a general reduction in performance status would be expected in a patient taking chronic pain medication.

Importantly, the purpose of the MBLS was not to prevent patients who require a surgery from being able to undergo one. It does not have a high positive predictive value (a characteristic it shares with the mFI-11 and the RPA classification) for death at 90 days, but it does have a high negative predictive value, suggesting that patients who are low risk by MBLS are predicted to have a greater than 3-month prognosis. Moreover, a low positive predictive value suggests that high-risk patients should not be excluded from surgery only based on their MBLS classification. On the contrary, we believe it should be a focal point of the overall treatment discussion, allowing the patient to decide for themselves based on their informed judgement. Risk-benefit analyses are complicated even for the most well-informed patients, and a score like the MBLS can help to provide additional context to their understanding of their disease. By providing more accessible risk assessment, the surgeon and oncologist can empower their patient to make the best possible decision and improve the patient-physician relationship.

### Limitations

This study has several limitations; first that it is a retrospective study, with inherent biases associated. Additionally, the patients were all treated at one tertiary cancer center albeit by several different surgeons. This leads to institutional bias and pipelines of care that may not be feasible or appropriate at other institutions. Next, treatment for metastatic cancer has evolved significantly, even since 2014, with the widespread adoption of immunotherapies and many clinical trials being offered at the study center. This leads to changes across the cohort timeline in treatment options available and expected postoperative course. Future directions and improvements for this work would be a larger dataset to allow for very granular looks at use of different cancer treatments and allotting points based on their use. Another limitation is the heterogeneous nature of the cohort, importantly that different malignant histologies may have different inherent factors which could be considered high risk. A score that included the histological diagnosis would be more predictive and individualized, and may be a good future direction for this work. Finally, score creation is inherently reductive, potentially removing factors which could be important to prognostication by virtue of narrowing the data.

## Conclusion

Brain metastasis requiring surgery is a unique disease state which requires individualized risk prognostication. Examining 548 patients, the authors investigated patients’ demographic and clinical characteristics and developed the six factor models. (Age, presence of supra and infratentorial metastases, hemorrhagic metastasis, hydrocephalus/severe ventricular compression, chronic opioid use, and poor functional status) to death at 90 days, creating the Metastatic Brain Lesion Severity Score (MBLS). This score predicted mortality robustly in a separate cohort of 318 patients for validation and outperformed the mFI-11 and RPA class in prognostication. Finally, it is a tool that can be used when discussing brain metastasis patients in multidisciplinary teams to quickly convey risk of surgery.

## Electronic supplementary material

Below is the link to the electronic supplementary material.

Supplementary Material 1

## Data Availability

No datasets were generated or analysed during the current study.
