# Supplementary material for: A novel lesion severity index to predict 90-day postoperative survival in brain metastasis patients
Source: J Neurooncol. 2025 Jun 11;174(3):799–808. doi: 10.1007/s11060-025-05109-7 (PMC12263791; doi:10.1007/s11060-025-05109-7)
Supplement: Supplementary file 1 — Supplementary Material 1 [file 11060_2025_5109_MOESM1_ESM.docx]

| **Supplementary Table 1** | **Creation Cohort** | | **Validation Cohort** | |
| --- | --- | --- | --- | --- |
| **Cancer Type** | **N** | **Percentage(%)** | **N** | **Percentage(%)** |
| **NSCLC** | 208 | 38.0 | 138 | 43.4 |
| **Melanoma** | 82 | 15.0 | 54 | 17.0 |
| **Renal Cell** | 36 | 6.6 | 17 | 5.3 |
| **HER2/HR Positive Breast Cancer** | 35 | 6.4 | 19 | 6.0 |
| **Triple Negative Breast Cancer** | 33 | 6.0 | 3 | 0.9 |
| **Colorectal/Anal Cancer** | 24 | 4.4 | 15 | 4.7 |
| **Any Sarcoma** | 20 | 3.7 | 11 | 3.5 |
| **Esophageal Cancer** | 18 | 3.3 | 14 | 4.4 |
| **Small Cell Lung Cancer** | 17 | 3.1 | 9 | 2.8 |
| **Gynecological Cancer** | 15 | 2.7 | 12 | 3.8 |
| **Urothelial cancer** | 10 | 1.8 | 9 | 2.8 |
| **HNSCC** | 9 | 1.6 | 6 | 1.9 |
| **Prostate cancer** | 8 | 1.4 | 2 | 0.6 |
| **Other GI Cancer** | 7 | 1.3 | n/a | n/a |
| **Neuroendocrine cancer** | 6 | 1.1 | 1 | 0.3 |
| **Other** | 20 | 3.6 | 8 | 2.6 |
| **Totals** | 548 | 100 | 318 | 100 |

Supplementary Table 1: Breakdown of individual cancer types in the collected cohorts. “Other” includes testicular cancer, choriocarcinoma, Merkel cell carcinoma, papillary thyroid cancer, salivary gland cancer, B cell lymphoma, squamous skin cell carcinoma, Hurthle cell carcinoma, and pheochromocytoma. GI = gastrointestinal; HR = hormone receptor; mL = milliliters; NSCLC = non-small cell lung cancer

| **Supplementary Table 2** | |  | | | | | | | | | | | | | | | | | |
| --- | --- | --- | --- | --- | --- | --- | --- | --- | --- | --- | --- | --- | --- | --- | --- | --- | --- | --- | --- |
| **Creation Cohort** | | **MBLS Score (No., %)** | | | | | | | | **Validation Cohort** | | **MBLS Score (No., %)** | | | | | | | |
|  |  | **0** | **1** | **2** | **3** | **4** | **5** | **6** | **7** |  |  | **0** | **1** | **2** | **3** | **4** | **5** | **6** | **7** |
| **Deceased at 90d** | **No** | 76 (93.8%) | 143 (84.1%) | 112 (84.2%) | 65 (69.9%) | 30 (57.7%) | 8 (50%) | 0 (0%) | 0 (0%) | **Deceased at 90d** | **No** | 41 (100%) | 69 (87.3%) | 66 (80.5%) | 31 (67.3%) | 26 (54.2%) | 7 (46.7%) | 1 (14.3%) | 0 |
|  | **Yes** | 5 (6.2%) | 27 (15.9%) | 21 (15.8%) | 28 (30.1%) | 22 (42.3%) | 8 (50%) | 2 (1000%) | 1 (100%) |  | **Yes** | 0 (0%) | 10 (12.7%) | 16 (19.5%) | 15 (32.7%) | 22 (45.8%) | 8 (53.3%) | 6 (85.7%) | 0 |
| **p=<0.001** | **Total** | 81 (14.8%) | 170 (31.0%) | 133 (24.3%) | 93 (17.0%) | 52 (9.5%) | 16 (2.9%) | 2 (0.4%) | 1 (0.2%) | **p=<0.001** | **Total** | 41 (12.9%) | 79 (24.8%) | 82 (25.8%) | 46 (14.5%) | 48 (15.1%) | 15 (4.7%) | 7 (2.2%) | 0 |
| **Creation Cohort** | | **MBLS = 3 or more** | | | | | |  | | **Validation Cohort** | | **MBLS = 3 or more** | | | | | |  | |
|  |  | Yes (N, %) | | No (N, %) | | **Totals** | |  |  |  |  | **Yes (N, %)** | | **No (No., %)** | | **Totals** | |  |  |
| **Deceased at 90 days** | **Yes** | 61 (37.2%) | | 53 (13.8%) | | 114 | |  |  | **Deceased at 90 days** | **Yes** | 51 (43.9%) | | 26 (22.9%) | | 77 | |  |  |
|  | **No** | 103 (62.8%) | | 331 (86.2%) | | 434 | |  |  |  | **No** | 65 (56.1%) | | 176 (87.1%) | | 241 | |  |  |
|  | **Total** | 164 | | 384 | | 548 | |  |  |  | **Total** | 116 | | 202 | | 318 | |  |  |
| **Odds Ratio for Death at 90 days (95% CI)** | | 3.699 (2.408 - 5.682) | | P-value | | <0.001 | |  |  | **Odds Ratio for Death at 90 days (95% CI)** | | 5.311 (3.060-9.218) | | P-value | | <0.001 | |  |  |
| **Creation Cohort** | | **MBLS = 4 or more** | | | | | |  |  | **Validation Cohort** | | **MBLS = 4 or more** | | | | | |  |  |
|  |  | **Yes (No., %)** | | **No (No., %)** | | **Totals** | |  |  |  |  | **Yes (N, %)** | | **No (No., %)** | | **Totals** | |  |  |
| **Deceased at 90 days** | **Yes** | 33 (46.5%) | | 81 (17.0%) | | 114 (20.8%) | |  |  | **Deceased at 90 days** | **Yes** | 38 (51.4%) | | 39 (16.0%) | | 77 | |  |  |
|  | **No** | 48 (53.5%) | | 396 (83.0%) | | 434 (79.2%) | |  |  |  | **No** | 36 (48.6%) | | 205 (84.0%) | | 241 | |  |  |
|  | **Total** | 71 (13.0%) | | 477 (87.0%) | | 548 | |  |  |  | **Total** | 74 | | 244 | | 318 | |  |  |
| **Odds Ratio for Death at 90 days (95% CI)** | | 4.246(2.514,7.170) | | P-value | | <0.001 | |  |  | **Odds Ratio for Death at 90 days (95% CI)** | | 5.548 (3.137-9.812) | | P-value | | <0.001 | |  |  |

**Supplementary Table 2:** Comparison of MBLS scoring and relation to death at 90 days between the creation and validation cohorts. Groupings of each total score and relative percentages are listed in the top portion. Both a cutoff of 3 and 4 were used as “high risk” for the purpose of comparison. CI = confidence interval; MBLS = Metastatic Brain Lesion Severity Score

| **Supplementary Table 3** | | | | | | | |
| --- | --- | --- | --- | --- | --- | --- | --- |
| **Grouped MFI score** | | | | **Grouped RPA Classification** | | | |
| **Frailty Level** | **Alive >90d** | **Deceased at 90d** | **p-value** | **RPA Class** | **Alive >90d** | **Deceased at 90d** | **p-value** |
| **Low Frailty (0-2 mFI-11)** | 350/432 (81.0%) | 82/432 (19.0%) | **0.043** | **Class 1** | 60/68 (88.2%) | 8/68 (11.8%) | **<0.001** |
|  |  |  |  | **Class 2** | 257/310 (82.9%) | 53/310 (17.1%) |  |
| **High Frailty (3+ mFI-11)** | 84/116 (72.4%) | 32/116 (27.6%) |  | **Class 3** | 117/170 (68.8%) | 53/170 (31.2%) |  |

**Supplementary Table 3:** Grouped MFI score and RPA classification for the creation cohort. MFI = modified frailty index; RPA = recursive partitioning analysis The p-value shows the chi-square test result for each classification score.

| ***Supplementary Table 4*** | **Creation Cohort Score Groupings (N, %)** | | | **Validation Cohort Score Groupings (N, %)** | | |
| --- | --- | --- | --- | --- | --- | --- |
| **MBLS Score** | 0-2 | 3-7 | | 0-2 | | 3-7 |
|  | 384/548 (70.1%) | 164/548 (29.9%) | | 202/318 (63.5%) | | 116/318 (36.5%) |
| ***Statistical Comparison in Creation Cohort*** | | | | | | |
| **Score** | **mFI-11 3 or greater** | | **RPA Class 3** | | **MBLS 3** | |
| **# patients labeled "high risk"** | **116/548 (21.2%)** | | **170/548 (31.0%)** | | **384/548 (29.9%)** | |
| **"Low risk" patients deceased at 90d** | **82/432 (19.0%)** | | **61/378 (16.1%)** | | **53/384 (13.8%)** | |
| **Sensitivity** | 28.0 | | 46.5 | | 53.5 | |
| **Specificity** | 80.6 | | 73.0 | | 76.3 | |
| **PPV** | 27.6 | | 31.2 | | 37.2 | |
| **NPV** | 81.0 | | 83.9 | | 86.2 | |
| **c-statistic** | **.514** | | **.609** | | **.649** | |
| **DeLong’s test (vs MBLS 3)** | **P=<0.001** | | **P=0.17** | | **n/a** | |

**Supplementary Table 4**: Statistical comparison of cohort groupings and head-to-head analysis with the modified frailty index. MBLS = Metastatic Brain Lesion Severity Score; mFI-11: modified frailty index-11; NPV = negative predictive value; PPV = positive predictive value; RPA = Recursive partitioning analysis
